# Collaborative Facilitation in Older Couples: Successful Joint Remembering Across Memory Tasks

**DOI:** 10.3389/fpsyg.2018.02385

**Published:** 2018-12-04

**Authors:** Amanda J. Barnier, Celia B. Harris, Thomas Morris, Greg Savage

**Affiliations:** ^1^Australian Research Council Centre of Excellence in Cognition and Its Disorders, Macquarie University, Sydney, NSW, Australia; ^2^Department of Cognitive Science, Macquarie University, Sydney, NSW, Australia; ^3^Dementia Centre, HammondCare, Greenwich, NSW, Australia; ^4^Department of Psychology, Macquarie University, Sydney, NSW, Australia

**Keywords:** memory, collaborative recall, collaborative facilitation, memory and aging, transactive memory, distributed cognition

## Abstract

Although we know a great deal about the effects of age on memory, we know less about how couples remember together and how day-to-day joint remembering might support memory performance. The possibility of memory support when couples remember together is in striking contrast with the standard finding from the collaborative recall literature that when younger pairs of strangers remember together they impair each other’s recall. In the current study, we examined the individual and joint remembering of 78 individuals who made up 39 older, long-married couples. We studied their performance on three memory tasks, varying in personal relevance: recalling a word list, listing all the countries in Europe, and remembering the names of their mutual friends. Couples gained clear collaborative benefits when they remembered together compared to when alone, especially European countries and mutual friends. Importantly, collaborative success was extremely stable over time, with good collaborators still successful 2 years later, suggesting that successful collaboration may be a stable couple-level difference. However, not all couples benefitted equally. Collaborative success related in part to particular conversational strategies that some couples, often those with discrepant individual abilities, used when collaborating. These findings highlight the value of analyzing individuals within their broader “memory systems” and the power of extending collaborative recall methods to more established intimate groups recalling a broader range of memory materials over longer time scales.

## Introduction

We live and age in a social context as members of multiple social groups. As individuals, we are members of couples, families, schools and universities, work teams and community groups. Wegner referred to these intimate, long-standing groups as “transactive memory systems” (Wegner et al., 1985; Wegner, 1987; see also Barnier et al., 2017), whereby individuals within such groups coordinate and share their cognitive resources. In doing so, “the group becomes capable of memory feats beyond that of any individual” (Wegner, 1987, p. 1; see also Barnier et al., 2017). Although the effects of aging on cognition typically are conceptualized and indexed at the level of the individual, considering individuals within their everyday social contexts – as members of transactive memory systems – may yield powerful insights into their day-to-day functioning (Harris et al., 2014a). For instance, Gerstorf et al. (2009) found that the cognitive trajectories of older married couples were linked, such that one partner’s decline could be predicted from the other. Indeed, Sommerlad et al. (2017) recently reported that married couples had a 42% lower risk of developing dementia compared to lifelong singletons and a 20% lower risk compared to bereaved individuals. These estimates were based on analysis of over 812,000 people involved in 15 aging and dementia studies around the world. These findings highlight the important cognitive and broader health benefits of intimate relationships as well as the value of considering people as part of their social groups in order to understand and predict their cognitive performance.

Research in a range of traditions suggests that remembering with intimate others can benefit memory performance, at least in the sense that the group outperforms a single individual. This benefit extends to older adults, even in the face of cognitive decline. For instance, Kemper et al. (1995) found that individuals with Alzheimer’s disease were able to recall more details from autobiographical memories when prompted by their spouse compared to when recalling alone. Specifically, spouses appear to use specific conversational strategies to help their partner with Alzheimer’s disease engage in genuinely joint collaborative reminiscing (Hydén, 2011). This can be so successful that collaborating with a spouse can eliminate the effects of baseline cognitive function on a semantic guessing task, such that those who are low in cognitive function are boosted to the level of those who are high (Rauers et al., 2010). Notably, the Rauers et al. (2010) study indicated that these benefits occurred when collaborating with an intimate partner but not with a stranger, perhaps because older spouses use more elaborations and fewer negative statements when they remember together compared to older strangers (Gagnon and Dixon, 2008).

These suggestions of “collaborative benefit” are important as they imply that remembering with other people may be an effective form of memory compensation (Dixon et al., 2001; Garrett et al., 2010). That is, collaboration with a close partner might provide an accessible and reliable tool to support individual memory in everyday life. Indeed, research suggests that people increasingly report relying on others as a compensation strategy as they age and as they experience cognitive decline (Dixon and de Frias, 2007). Cognitive psychologists have argued that collaboration with others may have therapeutic value in facilitating subsequent individual memory performance (Blumen et al., 2013; but see Barnier et al., 2013; Dixon, 2013 for implications of current gaps between the “lab and the world”).

However, possible benefits of shared remembering stand in stark contrast with the dominant finding of experimental cognitive psychology’s “collaborative recall” paradigm. In this literature, collaborating groups are compared not to single individuals but to the same number of individuals recalling alone who are pooled to form a “nominal group” (Basden et al., 1997; Weldon and Bellinger, 1997). The nominal group comparison is valuable as an index of the theoretical (additive) potential of all the individual members of a collaborative group (Weldon and Bellinger, 1997; see also Barnier et al., 2017). A remarkably robust literature shows that collaborative groups typically do not perform to their potential. Instead, they recall less than nominal groups, an effect known as “collaborative inhibition” (Harris et al., 2008; Rajaram and Pereira-Pasarin, 2010; Rajaram, 2011; Marion and Thorley, 2016). Collaborative inhibition occurs for both younger and older adults to a similar extent (Meade and Roediger, 2009; Henkel and Rajaram, 2011), and has been found for a range of materials including word lists, stories, and photos (for a review, see Marion and Thorley, 2016). Notably, collaborative inhibition is strongest and most robust for free recall tasks for which group members are more likely to have divergent individual retrieval strategies. However, a meta-analysis indicates that collaborative inhibition still occurs for “fixed order” memory tasks such as cued recall and recognition (see Marion and Thorley, 2016).

In the collaborative recall literature there are few exceptions to this robust collaborative inhibition effect in free recall. However, evidence is emerging that in select groups or for certain kinds of memories, collaboration can lead to “collaborative facilitation” instead of collaborative inhibition, such that a collaborative group recalls as much as, or even more than, a nominal group. For instance, Johansson et al. (2005) found that while on average older couples exhibited collaborative inhibition, a subset of couples – those with evidence of a transactive memory system as measured by high division of responsibility and high agreement – did not. Meade et al. (2009) found that a special kind of expert group – experienced pilots who are trained in effective communication – showed collaborative facilitation instead of inhibition when recalling information relevant to their domain of expertise. And in a small study directly relevant to the present study involving 12 older, long-married couples, Harris et al. (2011) found that some couples showed facilitation and some showed inhibition in recalling the names of their social club. So there is something beneficial in being a member of certain groups, consistent with work by Barnier et al. (2014) and Harris et al. (2017) who reported that older couples facilitated each other’s recall of specific episodic details about autobiographical memories (but see Ross et al., 2004). But these benefits of collaboration are not due to relationship alone. Instead, there is early evidence that the memory strategies couples use when they recall together influence successful collaboration, specifically, their use of cuing and repetition, and their avoidance of corrections and disagreements (Harris et al., 2011). The potential importance of effective communication strategies is highlighted also by research on collaboration in eyewitnesses: although pairs of eyewitnesses on average show a collaborative inhibition effect, those who use more communication strategies such as repetitions, re-statements, and elaborations recall more (Vredeveldt et al., 2016, 2017).

Overall, these findings suggest that collaborative facilitation may arise and persist within intimate and longstanding relationships in which effective communication strategies are adopted, just as predicted by transactive memory theory (Wegner et al., 1985; Wegner, 1987; Barnier et al., 2017). But so far there is only piecemeal evidence for collaborative benefits, and often from small studies, with different kinds of memory tasks and different approaches in terms of forming an individual control condition. Moreover, although individual differences appear important in the benefits of collaborative recall – such that even among intimate groups some couples collaborate effectively and others do not – so far, no research has tested the stability of collaborative success and whether the same couples who collaborate well on one occasion collaborate well on another. Finally, although seeking assistance from others as a form of memory compensation increases with need (i.e., with age and with cognitive decline; Dixon and de Frias, 2007) we do not yet know how success of collaboration is influenced by the relative abilities of individuals within the collaborating group. We aimed to extend prior research with a larger study of older couples, collaborating on a range of different recall tasks, and followed up over time to determine the extent to which collaborative success is stable.

In the current study we systematically tested the outcomes of collaborative recall in highly intimate groups – namely older, long-married couples – relative to their pooled, nominal group performance. These couples were well characterized in terms of their individual psychological and cognitive characteristics; they had been tracked for more than 10 years as part of a longitudinal aging project. We tested them on three tasks, indexing both personal and non-personal, semantic and episodic memory. We tested them in their homes, individually on an initial session and then collaboratively one week later. We returned approximately 24 months later and tested them collaboratively again. We aimed to examine: (1) their individual memory performance as well as the relationship between husbands’ and wives’ cognitive ability and individual memory performance; (2) collaborative performance, and any collaborative benefits over and above pooled individual performance; (3) stability of collaborative performance over time; and (4) correlates of collaborative benefit. In doing so, we aimed to determine whether collaboration with a spouse provides (long-term) benefits for memory, and what characteristics of couples and their collaboration are associated with these benefits.

## Materials and Methods

### Participants

We recruited participants from the Australian Imaging, Biomarkers, and Lifestyle (AIBL) Study of Ageing, a longitudinal study of community-dwelling individuals over the age of 60 who live in Melbourne or Perth, Australia. The AIBL Study was established in 2006 with 1,112 individuals recruited during the baseline phase. They underwent a screening interview, cognitive and mood assessments, blood-based biomarker analyses, and completed health and lifestyle questionnaires. Approximately a quarter of the sample underwent brain imaging, including magnetic resonance imaging (MRI) and Pittsburgh compound B – positron emission tomography (PiB-PET). A clinical review panel considered all medical, psychiatric, neuropsychological, and health data and classified 768 as healthy controls, 133 as having Mild Cognitive Impairment (MCI; Petersen et al., 1999; Winblad et al., 2004), and 211 as having Alzheimer’s disease (McKhann et al., 1984). Follow-up assessments of participants have occurred approximately every 18 months, with Wave 4 testing in 2014, just before we first met them and 54 months following initial baseline testing. Further details of the study and baseline characteristics are reported in Ellis et al. (2009).

For the current study, we were given access to a subset of healthy control participants from the larger AIBL study. We identified 94 participants (47 married couples) where both members of the couple were classified in their most recent AIBL assessment (typically Wave 4) as healthy controls. We first contacted couples via a letter inviting them to participate and confirmed their interest by telephone. Seventy-eight individuals (39 couples) were interested and available to participate and these were our participants for the current study. These 78 individuals (39 female, 39 male) were aged 68–90 years (*M* = 74.74, *SD* = 5.10), with 6–23 years of formal education (*M* = 14.47, *SD* = 4.11), and had been married 13–65 years (*M* = 49.46, *SD* = 8.78). The time from participants’ last neuropsychological assessment with AIBL was 1.36–3.13 years (*M* = 2.26*, SD* = 0.40). At their last AIBL assessment, participants had a Clinical Dementia Rating (CDR; Morris, 1997) of 0 confirming their healthy status (and consistent with our on-the-day testing detailed in the next section). Participants were considered by AIBL to have subjective memory complaints (SMC) if they were cognitively healthy as per this CDR criterion but answered yes to the question “Do you have difficulties with your memory?” at their last AIBL assessment. For the Word List task, one couple was not given the task, and one couple was inadvertently given different lists, so the analyses are based on *n* = 37 couples. For European Countries and Mutual Friends tasks, all couples completed the tasks and the analyses are based on *n* = 39 couples.

For Session 3, we returned 2 years later to 32 of our original 39 couples. For the remaining seven couples, one member of four couples was deceased or had experienced neurological illness in the interim, one couple no longer wished to participate, and two couples were uncontactable. At the time of Session 3, these 64 individuals (32 female, 32 male) were aged 70–92 years (*M* = 76.67, *SD* = 4.65), and had been married 40–67 years (*M* = 52.48, *SD* = 6.00). All remained cognitively healthy (as per our on-the-day testing detailed in the next section) except for one participant who self-reported having been diagnosed with mild dementia between Session 2 and Session 3. We retained his data to allow comparison back to Session 2 and also since analyses with and without his data yielded similar results. For the Word List, European Countries and Mutual Friends tasks, *n* = 32 couples. This study received ethics approval from Macquarie University’s Human Research Ethics Committee as well as approval from the Management Committee of the AIBL study.

### Materials

We used the following materials for the three memory tasks and three questionnaires administered, analyzed, and interpreted for this present paper (which were part of a larger set of personal and non-personal memory tasks, cognitive measures and questionnaires).

#### Word List Task

We adapted the stimuli and the procedure for the Word List task from the Hopkins Verbal Learning Test-Revised (HVLT-R: Brandt, 1991; Benedict et al., 1998). The HVLT–R is a list-learning task of episodic memory and consists of six alternate-form lists of 12 words, each comprising four words from three separate categories. These lists are normed for equivalence and for use as a clinical tool. Because of potential ceiling effects and based on previous research (e.g., Harris et al., 2011), we combined two equivalent lists from the HVLT-R (4 words each from 6 categories). For the purposes of the present study, we created three such modified lists, one by combining Form 1 and Form 4 (*List A*), one by combining Form 5 and Form 6 (*List B*), and the last by combining Form 2 and Form 3 *(List C)*.

#### The Mini-Mental State Examination

We administered the Mini-Mental State Examination (MMSE; Folstein et al., 1975) on the days of testing for Session 1 and Session 3. It also was administered in participants’ last AIBL assessment. The MMSE is a brief screen of general cognitive ability frequently used to discount an overt underlying dementia. The measure includes items investigating orientation, registration, attention, recall, language and visuospatial ability. The MMSE is scored out of 30 and a score of 24 or above often is interpreted as an indicator of healthy cognition (depending on the study and samples). Participants had a mean MMSE score of 28.87 (SD = 1.48) on the day of testing for Session 1 and a mean score 29.31 (SD = 1.25) on the day of testing for Session 3 approximately 2 years later. These scores were consistent with a mean MMSE score of 29.14 (SD = 0.88) during their last AIBL assessment, confirming their status as predominantly cognitively healthy.

#### The Geriatric Depression Scale - Short Form

We administered the Geriatric Depression Scale – Short Form (GDS-SF; Yesavage and Sheikh, 1986) on the days of testing for Session 1 and Session 3. It also was administered in participants’ last AIBL assessment. The GDS measures depressive symptomatology and includes 15 “yes or no” questions about how the participant felt over the last week such as “Do you feel that your life is empty?” and “Do you feel that your situation is helpless?”. The GDS-SF is scored out of 15 and a score 6 or above is interpreted as an indicator of depressive symptomology warranting further medical or psychiatric investigation. Participants had a mean GDS score of 1.08 (*SD* = 1.38) on the day of testing for Session 1 and a mean score of 0.98 (*SD* = 1.54) on the day of testing for Session 3 approximately 2 years later. These scores were consistent with a mean GDS score 0.93 (*SD* = 1.50) during their last AIBL assessment, confirming the absence of significant depressive symptomatology in our sample.

#### Personal Assessment of Intimacy in Relationships (PAIR)

We administered the Personal Assessment of Intimacy in Relationships Scale (PAIR; Schaefer and Olson, 1981) via mail prior to Session 1 and collected (or helped participants to complete and then collected) the questionnaire at the beginning of Session 1. The PAIR is a measure of couple intimacy and includes 30-items across 5 sub-scales: Emotional Intimacy (6 items), Social Intimacy (6 items), Intellectual Intimacy (6 items), Recreational Intimacy (6 items), and Sexual Intimacy (6 items). Participants rate how much a given statement applies to their current romantic relationship on a 5-point Likert scale (1 = *strongly disagree*; 5 = *strongly agree*). For participants’ privacy, we did not administer the Sexual Intimacy subscale in our study. Each of the four remaining subscales of the PAIR was scored out of 30 and a total was obtained by summing across subscales with a maximum score of 120. Husbands had a mean PAIR score of 92.77 (*SD* = 11.58) at Session 1 and a mean score of 95.55 (*SD* = 11.24) at Session 3. Wives had a mean PAIR score of 90.64 (*SD* = 13.10) at Session 1 and a mean score of 92.13 (*SD* = 13.45) at Session 3. These scores suggest a high level of intimacy across couples, albeit with some individual differences. Scores of the two partners within couples were significantly correlated, *r* = 0.44, *p* = 0.005.

### Procedure

We tested participants at their place of residence in two initial sessions, 1 week apart. For the subset of participants described above, we conducted a third session in their homes approximately 2 years later. Each session lasted approximately 1.5 h. We did not pay participants for their involvement in the study, but instead provided morning or afternoon tea.

#### Session 1

In Session 1, two experimenters tested participants simultaneously but individually in separate rooms of the couple’s house. Experimenters administered the Word List task using List A (as described above), modeling their procedure on the standard instructions of the HVLT-R, with the exception that the list contained 24 rather than 12 words (Brandt, 1991; Benedict et al., 1998). Experimenters read aloud the 24 items to individual participants at the rate of one word every 2 s. After each of three presentations of the list, experimenters instructed participants to immediately recall aloud as many words as they could remember. After the three presentation-recall cycles (Trials 1–3), there was a 20-min delay before the experimenters asked participants to recall the words again (Trial 4). Below we analyze and report Trials 1 and 4 for the Word List task. Trial 1 represents individuals’ and couples’ first attempt to learn and recall the word list whereas Trial 4 represents their final attempt after multiple opportunities to develop and implement memory strategies.

During the 20-min delay between Trials 3 and 4 of the Word List task, experimenters administered two additional memory tasks. First, the European Countries task, a non-personal semantic recall task, in which they asked participants to recall as many European countries as possible within a 2-min time limit. Specifically, the experimenters instructed participants: “I want you to tell me as many countries in Europe as you can think of. Try to make your list as long as possible. You will have 2 min to do this task. Are you ready? Okay, go.” Following this, participants completed the Mutual Friends task, a personal semantic recall task, in which the experimenters asked participants to recall as many mutual friends or acquaintances as possible within a 2-min time limit. Specifically, the experimenters instructed participants: “I would like you to tell me all of the mutual friends or acquaintances both you and [Spouse’s name] know. Please only tell me people for whom you know both their first name and last name. Also, please do not include family members in your list. Try to make your list as long as possible. You will have 2 min to do this task. Are you ready? Okay, go.” We limited participants to 2 min for each of these individual recall tasks because they were more open ended than the Word List task, which had a maximum of just 24 items. In this way, we kept the time for each task relatively constant across participants. If recall appeared blocked, the experimenters prompted participants to continue trying to think of additional items until 2 min had elapsed. Finally, the experimenters asked participants to recall in detail a number of autobiographical memories, the results of which we do not report here. At the end of Session 1 participants completed the MMSE and GDS.

#### Session 2

One week after the initial individual recall session, the two experimenters returned to each couple’s house and tested participants together in a collaborative recall session. Experimenters administered the Word List task using List B (as described above) and the same procedure as in Session 1 of four recall tests but with an instruction for participants to work together to jointly recall the words. The experimenters did not instruct participants on how to collaborate or how to resolve disagreements, except that they should “work together to help each other to recall as many items as possible.” During the 20-min delay between Trials 3 and 4 of the Word List task, experimenters administered the European Countries task and the Mutual Friends task, again with the same instructions as in Session 1 except that couples should “work together to tell me as many countries in Europe as you can think of” and “work together to tell me all of the mutual friends or acquaintances that you both know.” We limited participants to 4 min for each of these joint recall tasks. Our aim was to equate the time each couple had during Session 1 with their collaborative recall in Session 2, since their individual Session 1 recall was pooled together to give each couple a “nominal” score (2 × 2 min = 4 min). If recall appeared blocked, the experimenters prompted participants to continue trying to think of additional items until 4 min had elapsed. As in Session 1, the experimenters asked participants to jointly recall in detail a number of autobiographical memories, the results of which we do not report here.

#### Session 3

Twenty-two to 32 months (*M* = 27.06, *SD* = 2.61) after Session 2, one of the original experimenters returned to each couple’s house and tested participants together in a final collaborative recall session. As in Session 2, the experimenter first administered the Word List task using List C (as described above) and the same procedure of four recall tests and an instruction to work together to jointly recall. During the 20-min delay between Trials 3 and 4 of the Word List task, the experimenter administered the European Countries task and the Mutual Friends task, again with the same instructions as in Session 2 for couples to “work together to tell me as many countries in Europe as you can think of” and “work together to tell me all of the mutual friends or acquaintances that you both know.” We again limited participants to 4 min for each of these joint recall tasks. If recall appeared blocked, the experimenter prompted participants to continue trying to think of additional items until 4 min had elapsed. At the end of Session 3, participants individually completed the MMSE and GDS, as in Session 1 approximately 2 years earlier.

### Coding and Scoring

Two trained research assistants transcribed all sessions in full from the audio recordings. We coded and scored each memory task for the total number of items (words, countries, friends) recalled and the use of strategies to assist recall.

#### Items Recalled

We calculated the total number of words correctly recalled for all trials of the Word List task; we considered a word correct if it corresponded with the studied list or was a close approximation (e.g., singular/plural errors were considered correct). We also calculated the total number of countries correctly recalled on the European Countries task; we considered a country correct if it corresponded with a United Nations list of European countries and was generated within the time limit for that task. Finally, we calculated the total number of friends correctly recalled on the Mutual Friends task; we considered a friend correct if it included both a first name and a surname and was generated within the time limit for that task.

#### Nominal Group Scores

For individual recall (Session 1) we calculated “nominal scores” for each couple on each task, as is standard in the collaborative recall literature (see Harris et al., 2008). Nominal scores consisted of pooling together the items recalled by the two individuals in the couple, only counting the redundant items once. We calculated nominal scores for couples on all three tasks, such that words, European countries, or names of mutual friends that were mentioned by both individuals on their separate individual recalls were only counted once in the nominal group score. Thus, nominal group scores represent the potential output of the two individuals combined, and this score is what collaborative scores can be compared to.

#### Strategy Use

We coded the collaborative (Sessions 2 and 3) transcripts of all tasks for the use of implicit and explicit memory recall strategies. We noted and counted the use (0 = not used, 1 = used once, 2 = used more than once) of four types of recall strategies: (1) *Implicit strategies*, where there was evidence of organization or chunking in recall, but where this was not explicitly named or discussed. For instance, we counted three items from the same category appearing in a row as evidence of an implicit strategy. This might include recalling words grouped by semantic category (as in the Word List task), words that started with the same letter, European countries that were geographically linked (e.g., England, Ireland, Scotland, Wales), or friends with the same first name. (2) *Mentioned strategies*, where there was evidence of vocalized strategy use, but where its strategic nature was not explicit. For instance, this included statements such as “There were more clothes…” in the Word List task, or “All those ex-communist countries…” in the European Countries task. (3) *Explicit categorical strategies*, where there was evidence of an explicit attempt to organize and cue recall using categories. For instance, this included statements such as “I’m thinking about the categories, there were clothes….” or “Let’s start with our church friends”. (4) *Explicit idiosyncratic strategies*, where there was evidence of an explicit attempt to organize and cue recall using some other organization or shared knowledge. For instance, this included using a story to structure word recall or using the alphabet to cue recall. Notably, in order to count as explicit, the strategy had to be explicitly stated such as “Let’s start with all the As.” An independent research assistant coded the 234 transcripts from the three tasks in Sessions 2 and 3 for the presence of these strategies and a second research assistant coded 26% of the transcripts. Their inter-rater reliability was *r* = 0.82. We retained the first research assistant’s coding following discussion of any disagreements.

## Results

First, we focus on participants’ individual Session 1 performance as well as the relationship, if any, between husbands’ and wives’ cognitive ability and individual memory performance. Second, we focus on the costs and benefits of remembering together across the three memory tasks by comparing Session 1 to Session 2. Third, we report couples’ collaborative recall 2 years later and look for evidence of stability in collaborative success and collaborative processes by comparing Session 1, Session 2, and Session 3. Finally, we explore correlates of successful collaboration, including features of the individuals, their relationship, and their collaborative memory strategies.

### Individual Memory Performance

Table 1 presents the mean number of words on Trials 1 and 4 (maximum score on each trial is 24), European countries (maximum score is 57), and mutual friends recalled by husbands and wives remembering alone in Session 1 as well as the range of their scores. Note here that the level of analysis is individuals not groups (*n* = 74 for the Word List task and *n* = 78 for the European Countries and Mutual Friends tasks). On the Word List task, participants generally improved across trials (see Table 1); a paired samples *t*-test confirmed that on average, they recalled more words on Trial 4 than Trial 1, *t*(73) = 14.02, *p* < 0.001. However, we saw considerable variability in individual performance; whereas some older adults remembered most of the words especially by Trial 4, others remembered few words even after four trials (see Table 1). Word List performance was unrelated to gender and memory complaint status, all *t*s < 1.40, all *p*s > 0.16, and unrelated to MMSE and GDS scores on the day of testing and length of the couple’s relationship, all *r*s < 0.16, all *p*s > 0.16. However, age negatively correlated with performance. Older participants remembered fewer words on Trial 1, *r* = -0.26, *p* = 0.026, but not on Trial 4, *r* = -0.18, *p* = 0.133. Years of education also marginally correlated with performance on Trial 1, *r* = 0.22, *p* = 0.070, and Trial 4, *r* = 0.23, *p* = 0.051.

**Table 1:** Individual performance across memory tasks in Session 1.

	Words Trial 1	Words Trial 4	European Countries	Mutual Friends
Individual scores	8.84 (3.38)	15.18 (5.06)	19.24 (7.42)	18.83 (9.22)
Range of scores	2 – 17	3 – 24	2 – 33	0 – 46


*For individual scores, values are mean number of items with standard deviations in parentheses.*

On the European Countries and Mutual Friends tasks, again we saw considerable variability in individual recall (see Table 1). Recall of European countries was unrelated to memory complaint status, *t*(76) = 0.36, *p* = 0.723, and unrelated to age, MMSE and GDS scores on the day of testing, and length of the couple’s relationship, all *r*s < 0.15, all *p*s > 0.200. However, men (*M* = 21.36, *SD* = 7.52) remembered more countries than women (*M* = 17.13, *SD* = 6.79), *t*(76) = 2.61, *p* = 0.011, and participants with more years of education remembered more countries, *r* = 0.42, *p* < 0.001. In contrast, recall of mutual friends was unrelated to memory complaint status and gender, all *t*s*(*76) < 0.67, all *p*s > 0.503, and unrelated to MMSE scores on the day of testing, length of the couple’s relationship, and years of education, all *r*s < 0.20, all *p*s > 0.076. However, older participants remembered fewer friends’ names, *r* = -0.29, *p* = 0.009, and people with higher depression scores on the day of testing also tended to remember fewer friends’ names, *r* = -0.22, *p* = 0.051 (see Table 1).

Thus, we saw clear differences in individual abilities and interesting patterns across the three memory tasks. For instance, although participants recalled both fewer words and friends, this may be for quite different reasons (such as a reduction in their social circle with age, which would impact the Mutual Friends task). We also saw evidence of expertise on the European Countries task, where men and those with more education (perhaps with greater exposure or opportunities to travel) recalled the names of more countries. Interestingly, whereas the recall performance of husbands and wives within each couple was not correlated for the Word List task Trial 1, *r* = 0.02, *p* = 0.891, and Word List task Trial 4, *r* = -0.05, *p* = 0.764, it was positively correlated for both the European Countries and Mutual Friends tasks, *r* = 0.43, *p* = 0.006 and *r* = 0.33, *p* = 0.038, respectively. In other words, although they worked alone on these latter two tasks, husbands and wives tended to both do well or both do poorly. However, we also noted discrepancies in individual performance or abilities. Husbands and wives within couples differed in their recall by 0–10 words (*M* = 4.05, *SD* = 2.40) on the Word List task Trial 1, by 0–20 words (*M* = 5.81, *SD* = 4.44) on the Word List task Trial 4, by 0–18 countries (*M* = 7.36, *SD* = 4.64) on the European Countries task, and by 0–22 friends (*M* = 8.82, *SD* = 6.05) on the Mutual Friends task, which suggests that in Week 2 some participants collaborated with spouses of either much greater or lower ability than their own across the different memory tasks. This variation is worth keeping in mind as we next analyze the costs and benefits when couples remembered together. We return to this issue of discrepancy later in the analyses.

### Costs and Benefits of Remembering Together

Table 2 presents the mean number of words on Trials 1 and 4, European countries, and mutual friends recalled by couples in Sessions 1 versus 2. For the following analyses, we examined the effects of collaboration at the level of couples, and thus we compared their pooled nominal performance at Session 1 to their collaborative performance at Session 2. To account for individual differences in productivity and the interrelatedness of husbands’ and wives’ Session 1 performance, couples acted as their own baseline; the effects of collaboration were measured against couples’ combined individual performance.

**Table 2:** Nominal and collaborative performance across memory tasks in Sessions 1 and 2.

Session	Words Trial 1	Words Trial 4	European Countries	Mutual Friends
Session 1 nominal	13.81 (3.07)	20.51 (3.02)	26.56 (6.68)	30.85 (12.34)
Session 2 collaborative	11.92 (3.42)	20.43 (3.14)	29.79 (7.52)	47.64 (17.99)
Collaborative benefit	-1.89 (2.88)	-0.08 (2.79)	3.23 (4.08)	16.79 (10.46)


*Values are mean number of items with standard deviations in parentheses.*

For the Word List task, a 2 (session: nominal vs. collaborative) × 2 (trial: Trial 1 vs. Trial 4) repeated measures ANOVA yielded significant main effects of session, *F*(1,36) = 6.23, *p* = 0.017, $\eta_{p}^{2}$ = 0.15, and trial, *F*(1,36) = 366.16, *p* < 0.001, $\eta_{p}^{2}$ = 0.91, as well as a significant interaction, *F*(1,36) = 30.33, *p* < 0.001, $\eta_{p}^{2}$ = 0.27. Overall, participants recalled more words in the nominal (*M* = 17.16, *SD* = 2.71) than the collaborative (*M* = 16.18, *SD* = 2.94) condition while recall improved considerably across the four trials (from *M* = 12.87, *SD* = 2.91 on Trial 1 to *M* = 20.47, *SD* = 2.75 on Trial 4). Interestingly, evidence of collaborative inhibition diminished across the trials as indicated by the interaction between condition and trial. Whereas couples recalled fewer words together than alone on Trial 1, *t*(36) = 3.99, *p* < 0.001, there were no differences in their nominal and collaborative recall on Trial 4, *t*(36) = 0.18, *p* = 0.861. This suggests that as couples got better at working together to recall more of the word list, the costs of collaboration diminished (see Table 2).

As importantly, we did not find overall collaborative costs on the European Countries and Mutual Friends tasks. Instead, couples showed significant collaborative facilitation. As can be seen in Table 2, couples correctly recalled more European countries together in Session 2 compared to their pooled nominal performance in Session 1, *t*(38) = 4.94, *p* < 0.001, and they recalled substantially more names of mutual friends together in Session 2 compared to their pooled nominal performance in Session 1, *t*(38) = 10.02, *p* < 0.001 (see Table 2). In other words, collaborative facilitation was both more likely and of greatest magnitude when couples recalled personally relevant, shared information.

We also calculated a “collaborative benefit” score for each task, defined as Session 2 collaborative scores minus Session 1 nominal scores (see Table 2). A positive collaborative benefit score indicates *collaborative facilitation* whereas a negative score indicates *collaborative inhibition*. This allowed us to characterize each couple in terms of the extent to which they benefited from collaboration. Collaborative benefit scores on Word List Trial 1 and Trial 4 were positively correlated, *r* = 0.43, *p* = 0.008, and collaborative benefit scores for the European Countries and Mutual Friends tasks also were positively correlated, *r* = 0.34, *p* = 0.033. But benefit scores for the European Countries and Mutual Friends tasks were not correlated with Word List Trial 1 or 4, all *r*s < 0.20, all *p*s > 0.24. In other words, couples who collaborated well on the Word List task showed collaborative success across trials, and couples who collaborated well on the European Countries task also collaborated well on the Mutual Friends task. This suggests that success on different tasks may rely on different processes, which we return to after considering first whether collaborative success is a stable characteristic.

### Stability of Collaborative Benefits and Strategies

To test stability of collaborative benefit, we returned to 32 of the original 39 couples and conducted a collaborative Session 3, approximately 2 years after they completed Sessions 1 and 2 (see Participants). First, for each of the four tasks, we compared couples’ Session 1 nominal scores to their Session 3 collaborative scores using pairwise *t*-tests (see Table 3). We found the same general pattern of results 2 years after we first tested couples, including evidence on some tasks of collaborative benefits. Specifically, we found collaborative inhibition for Word List Trial 1 (where Session 3 scores were lower than Session 1 scores), *t*(29) = 2.50, *p* = 0.018, but no difference for Word List Trial 4, *t*(29) = 1.95, *p* = 0.060. And we found collaborative facilitation for both the European Countries and Mutual Friends tasks (where Session 3 scores were significantly higher than Session 1 scores), *t*(31) = 4.10, *p* < 0.001 and *t*(31) = 16.81, *p* < 0.001. Although couples remembered slightly fewer mutual friends in Session 3 compared to Session 2, *t*(31) = 2.82, *p* = 0.008, their overall performance on Session 3 was very similar to Session 2 2 years earlier, with no significant differences on the other three tasks [Word List Trial 1: *t*(29) = 0.36, *p* = 0.721; Word List Trial 4: *t*(29) = 1.72, *p* = 0.096; European Countries task: *t*(31) = 0.04, *p* = 0.965]. Overall, in Session 3 we still observed clear benefits of remembering together on the more personal tasks (see Table 3).

**Table 3:** Nominal and collaborative performance across memory tasks in Sessions 1, 2, and 3 for couples who completed all three sessions.

Session	Words Trial 1	Words Trial 4	European Countries	Mutual Friends
Session 1 nominal	14.07 (2.94)	20.73 (2.95)	27.28 (6.37)	33.56 (11.33)
Session 2 collaborative	12.27 (3.37)	20.57 (2.88)	30.16 (7.38)	51.25 (16.16)
Session 3 collaborative	12.53 (3.73)	19.57 (3.22)	30.18 (7.35)	46.41 (13.85)
Collaborative benefit	-1.53 (3.36)	-1.17 (3.27)	2.91 (4.01)	12.84 (8.68)


*Values are means with standard deviations in parentheses. Collaborative Benefit scores are calculated as Session 3 Collaborative – Session 1 Nominal.*

When we calculated collaborative benefit scores for Session 3 (as the difference between collaborative scores on this session and nominal scores on Session 1, see Table 3), they positively correlated with Session 2 collaborative benefit scores (see Table 2) for Word List Trial 4, *r* = 0.46, *p* = 0.010, the European Countries task, *r* = 0.53, *p* = 0.002, and Mutual Friends task, *r* = 0.50, *p* = 0.004 (but not for Word List Trial 1, *r* = 0.17, *p* = 0.36). This suggests that collaborative benefits are a stable, individual difference characteristic of couples and that some couples remain more effective collaborators than others even when tested 2 years later.

#### Correlates of Successful Collaboration

What accounts for these (stable) benefits of collaboration? We explored whether couples’ collaborative benefit scores across the Word List, European Countries, and Mutual Friends tasks in Session 2 were associated with husbands’ and wives’ demographic variables (age, years of education, relationship length), mood and cognitive status (GDS and MMSE), and intimacy scores (PAIR). For the demographic variables, only wives’ years of education positively correlated with the collaborative benefit score on Word List Trial 4, *r* = 0.34, *p* = 0.044; all other correlations were not significant, all *r*s < 0.26, all *p*s > 12. Both husbands’ and wives’ MMSE and GDS scores were not correlated with benefit scores, all *r*s < 0.21, all *p*s > 0.20. Finally, husbands’ and wives’ intimacy scores, indexed by total PAIR scores, were not associated with collaborative performance across tasks, all *r*s < 0.27, all *p*s > 0.10. Thus, our individual difference measures did not appear to explain differences in collaborative performance, perhaps because this comparison between individual level data (of husbands or wives) and group level data (of benefit scores) is insensitive.

We characterized couples in terms of discrepancies between the individuals within each couple. That is, to what extent were wives and husbands similar or different in their baseline recall? We gave each couple a “discrepancy score” on each of the recall tasks, operationalized as the absolute difference in the number of items recalled by the two individuals when they remembered alone. A higher discrepancy score indicates that one member of the couple recalled many more items when alone than the other, while a lower discrepancy score indicates more equal individual performance. For each task in Session 2, we looked for relationships between discrepancy and collaborative benefit. But, we found no relationships, all *r*s < 0.20, all *p*s > 0.22.

Next we coded for evidence of implicit, mentioned, explicit categorical, and explicit idiosyncratic memory strategies in the transcripts from Session 2, as described in the Method above. We then calculated strategy scores for each couple. Couples were assigned one point each for: (1) implicit strategy use; (2) implicit strategy use more than once; (3) mentioned strategy use; (4) mentioned strategy use more than once; (5) explicit categorical strategy use; (6) explicit categorical strategy use more than once; (7) explicit idiosyncratic strategy use; (8) explicit idiosyncratic use more than once; (9) explicit categorical use acknowledged and picked up by the partner; and (10) explicit idiosyncratic strategy use acknowledged and picked up by the partner. Thus, couples received a strategy score out of 10.

Couples’ average strategy scores in Session 2 were: 2.92 (*SD* = 1.96) on Word List Trial 1, 4.21 (*SD* = 1.44) on Word List Trial 4, 3.56 (*SD* = 1.55) on the European Countries Task and 3.59 (*SD* = 1.76) on the Friends Task. A 4-level (task) repeated measures ANOVA indicated that scores differed across tasks, *F*(3,111) = 5.16, *p* = 0.005, $\eta_{p}^{2}$ = 0.12. Follow-up pairwise comparisons (with Bonferroni adjustment for multiple comparisons) indicated that strategy scores for the Word List Trial 4 were significantly higher than those for Word List Trial 1, *p* < 0.001, but no other significant differences between tasks, all *p*s > 0.15. Overall, strategies developed with experience on the Word List task.

We were interested in whether the use of strategies during collaboration, indicated by these scores, helped to explain collaborative benefits in Session 2. Collaborative strategy scores positively correlated with collaborative benefits for Word List Trial 1, *r* = 0.35, *p* = 0.036, but not for Word List Trial 4, *r* = 0.18, *p* = 0.293. Interestingly, couples who used more strategies at Trial 1 still had higher benefit scores at Trial 4, *r* = 0.44, *p* = 0.007. Collaborative strategy scores also positively correlated with collaborative benefits for the Mutual Friends task *r* = 0.37, *p* = 0.020, but not the European Countries task, *r* = 0.15, *p* = 0.351. Thus, strategy use seemed to be associated with collaborative memory benefits over and above baseline individual recall, but only on certain tasks. This likely reflects, at least in part, the nature of each task. For instance, whereas 56% of couples used explicit idiosyncratic strategies when collaborating to recall European countries (e.g., “Let’s start with the places we went on our trip”), only 10% used explicit categorical strategies on this task (e.g., “Let’s start with Eastern Europe”). In contrast, 49% of couples used explicit categorical strategies when collaborating to recall friends and acquaintances (e.g., “Let’s start with my work people and then we’ll do yours”), whereas only 28% used explicit idiosyncratic strategies on this task (e.g., “Let’s start north and then come down the coast”). Thus, the different tasks lent themselves to different strategies.

Finally, we tested whether some couples were more likely to use strategies than others; specifically, we tested whether there was a relationship between discrepancy in the abilities within the couple and their use of strategies in Session 2. We found a positive correlation between discrepancy and strategy score for the Mutual Friends task, *r* = 0.36, *p* = 0.025, but not for the other tasks, all *r*s < 0.25, all *p*s > 0.13. Thus, when recalling the names of mutual friends, couples who started out with a large discrepancy in their (Session 1) individual ability to recall friends’ names appeared to use implicit and explicit strategies when doing this task together.

We coded and calculated collaborative strategy scores for Session 3 in the same way as for Session 2 above. Strategy scores from Session 2 and Session 3 correlated for all four tasks: Word List Trial 1, *r* = 0.42, *p* = 0.022, Word List Trial 4, *r* = 0.35, *p* = 0.062, European Countries, *r* = 0.44, *p* = 0.012, and Mutual Friends, *r* = 0.65, *p* < 0.001, indicating that those couples who used more strategies when collaborating in Session 2 still used more strategies when collaborating 2 years later in Session 3. Notably, Session 3 strategy scores and Session 3 collaborative benefit scores positively correlated, much like during Session 2, for Word List Trial 1, *r* = 0.44, *p* = 0.016, and marginally for the Mutual Friends task, *r* = 0.31, *p* = 0.079 (but not for Word List Trial 4 or for the European Countries task, all *r*s < 0.13, all *p*s > 0.477). In other words, using more strategies again appeared to lead to better recall on some, but not all, tasks. Finally, discrepancies in individual performance on Session 1 2 years earlier positively correlated with strategy use on the Mutual Friends task in Session 3 (just as in Session 2), *r* = 0.39, *p* = 0.027 (but not for the other tasks, all *r*s < 0.16, all *p*s > 0.38). Although caution is warranted due to the correlational nature of this analysis and multiple comparisons, the overall pattern suggested that, at least on some tasks, the use of strategies was associated with collaborative benefits, couples with greater discrepancies used more strategies, and strategy use remained reasonably stable over time.

## Discussion

Our findings point to clear collaborative benefits when older adults in longstanding, intimate relationships remember together compared to alone. These benefits represent not just an elimination of the usual collaborative inhibition seen in most collaborative recall studies (e.g., Basden et al., 1997; Weldon and Bellinger, 1997; for reviews see Harris et al., 2008; Rajaram, 2011), but genuinely emergent outcomes where couples perform together in ways that are literally “more than the sum of their parts” (Wegner et al., 1985; Wegner, 1987; Harris et al., 2011, 2014a; Barnier et al., 2017). This study is one of only a few that have demonstrated collaborative facilitation rather than collaborative inhibition (e.g., Meade et al., 2009; Harris et al., 2011, 2017) and our results are especially notable because we see strong benefits of collaborative remembering in older adults; our participants ranged in age from 70 to 92 years old by Session 3. One key to collaborative facilitation seems to be real world groups recalling personally significant material during conversations that utilize their everyday communicative strategies (Barnier et al., 2014; Harris et al., 2014a; see also Johansson et al., 2005; Gagnon and Dixon, 2008). Only in such cases might we see collaborative facilitation, which until now has proven relatively elusive in the laboratory (Barnier et al., 2008).

Our findings indicate also that collaborative benefits may be strongly task dependent. Whereas we appeared to eliminate collaborative inhibition from Word List Trial 1 to Trial 4 (where overall performance moved from collaboration inhibition to no evidence of inhibition or facilitation), we only observed collaborative facilitation for the more personally relevant European Countries and Mutual Friends tasks. The magnitude of this facilitation was greatest for the Mutual Friends task where husbands and wives relied on their shared histories and knowledge to successfully collaborate in generating a list of first and last names. We also noticed evidence of expertise, especially in the European Countries task. Husbands typically were better at this task when remembering alone in Session 1 and often contributed more to joint remembering in Session 2. This may have been because they traveled more in their working lives, served overseas in the military, organized the couples’ vacations or held a job that involved greater knowledge in this area (e.g., being a Geography teacher as one husband told us). Such patterns of integrated versus differentiated knowledge provide empirical observations of Wegner’s (1987) theory of transactive memory, where encoding, storage and retrieval of knowledge is distributed across the members of established, intimate groups (see also Wegner et al., 1985; Barnier et al., 2017). In everyday life, husbands and wives often take responsibility for remembering different aspects of their lives as well as remembering things in common.

Consistent with other research we have conducted (e.g., Harris et al., 2011; for review see Harris et al., 2014a), the ways in which couples communicated what they knew to each other was important, especially for some tasks. On the Mutual Friends task, for instance, those couples who agreed on and used memory strategies to coordinate their knowledge remembered more names than couples who did not mention or use memory strategies. Memory collaboration may be more successful when couples can “get on the same page” and stay on it together. These results support Wegner’s (1987) proposal that within an effective transactive memory system, members must hold knowledge relevant to the task, know what other members of the group know, and be able to communicate it effectively with one another. However, as already noted, the operation of such a system is nuanced by the nature of the task and individual expertise. Sometimes one member of the group may be most able to complete the task or the majority of the task, such as listing all the countries in Europe, without the input of other members of the group. It is an interesting question, however, whether groups consider memory collaborations dominated by one person “successful.” Although memory researchers might count an equal or greater number of items recalled together versus alone as “success,” individual members or a couple may not (Barnier et al., 2013, 2016), emphasizing that remembering together serves functions well beyond mere productivity (Harris et al., 2014b).

This experiment also offers the first evidence of the long-term stability of collaborative success and strategies. Whereas almost all collaborative recall studies test collaborative recall within single sessions or over short time frames (Harris et al., 2008; Rajaram, 2011), we returned to test our long-married couples on average 2 years after their first collaborative session. We found that successful collaborators in Session 2 were still collaborating successfully 2 years later, despite the different tasks, passage of time, and potentially declining cognition. Those couples who relied on more implicit and explicit memory strategies during joint remembering in Session 2 used similar strategies 2 years later. However, not all couples appeared to use such strategies even though they are predictive of collaborative success, at least for some tasks. Thus, successful collaborative remembering using sensitive communication may be conceptualized as a stable skill that particular couples have developed. Understanding the variables that underlie the development of such skills, and whether such skills can be taught or encouraged via intervention, are important directions for future research.

We noted individual differences between couples in the extent to which they benefited from collaboration, although we did not clearly identify factors associated with these differences such as age or intimacy. Analysis of the individual memory performance of husbands and wives working alone in Session 1 revealed a range of discrepancies in memory performance or ability within married couples. In other words, not all of the groups were composed of individuals with similar abilities, inconsistent with the unspoken assumption of the collaborative recall literature (where groups often are assumed to be equal). At least for some of our tasks, this discrepancy in the individual abilities of husbands and wives influenced the likelihood of them using more memory strategies as they remembered together. In turn, for some tasks, this use of more memory strategies predicted greater collaborative benefits. This suggests a link between the individual abilities of group members, a potential need that one member might have for scaffolding or assistance from their partner, sensitive awareness of this need by their partner, the use of conversational tactics as they worked together to support one another’s recall, and finally collaborative success (see also Barnier et al., 2008, 2014; Gagnon and Dixon, 2008; Rauers et al., 2010; Hydén, 2011; Harris et al., 2014a). This pathway from individual ability to collaborative success could be tested more directly in future research with groups where one partner has greater cognitive need, such as in married couples where one partner has cognitive decline or dementia.

There are two alternative explanations for our findings of collaborative benefits. First, it is possible that improvements from (nominal) Session 1 to (collaborative) Session 2 simply were the result of practice effects. We tested all participants individually in the first session and collaboratively in the second session rather than counterbalancing recall conditions because research indicates that post-collaborative individual recall is not equivalent to pre-collaborative individual recall (due to post-collaborative benefits; for a review see Marion and Thorley, 2016). However, the fact that patterns of collaboration were similar 2 years later rules out practice effects as an adequate explanation since there was no initial individual recall in Session 3. Our more exploratory finding that collaborative benefits were associated with strategy use, at least on some tasks, also suggests that practice effects were not driving these findings.

Second, it is possible that improvements from Session 1 to Session 2 were influenced by our choice to limit individual recall to 2 min compared to collaborative recall of 4 min. We made this choice because when Session 1 individual recall was pooled together, couples had 4 min in total just as they had 4 min in total in Session 2 when they collaborated. Perhaps we inadvertently cut off participants in Session 1 with our 2-min limit? But this was not the case: analysis of the time course of recall indicated that the most participants in the individual (88.46% in the European Countries task and 71.79% in the Mutual Friends task) had ceased to recall items before the time limit elapsed. If anything, the time limits impacted collaboration more than individual recall: while most couples ceased recalling items before the time limit when they collaborated on the European Countries task (94.87%), fewer couples ceased to recall within the time limit on the Mutual Friends task (43.59%). Moreover, even when we examined Session 2 collaborative recall scores by only counting items recalled within the first 2 min – the most stringent comparison possible – couples already were numerically higher than their Session 1 individual scores on both the European Countries and Mutual Friends tasks. And when we analyzed only the subset of couples in which both individuals had ceased to recall items within the 2-min time limit, significant collaborative facilitation remained on both the European Countries and Mutual Friends tasks (see Hyman et al., 2013, for a similar analysis and detailed discussion of timing in collaborative recall). Thus, collaborative facilitation was not driven by a lack of opportunity to complete the individual recall task, and our choice of time limits cannot explain collaborative facilitation on the European Countries and Mutual Friends tasks.

Our findings demonstrate that collaboration can benefit remembering of long married couples, at least on some tasks, and that successful collaboration is a stable skill over quite long periods, at least for some couples. Although most collaborative research has found clear evidence of individual benefits post collaboration (for a review, see Harris et al., 2008; Rajaram, 2011), in this paper we report some of the only clear evidence for benefits during collaboration (see also Meade et al., 2009; Harris et al., 2011, 2017). This widens further the potential practical applications of collaborative recall (Barnier et al., 2013; Blumen et al., 2013; Dixon, 2013), since collaboration may be especially valuable as we age and need external memory support (Barnier et al., 2014). Importantly, however, not everyone benefited from collaboration with their spouse. As in previous research (Harris et al., 2011), underneath our group level effects, some couples collaborated successfully whereas others were less successful. It is both theoretically and practically important that we understand the source and nature of these individual differences, especially if we hope to extract lessons from collaborative recall research and implement it in memory interventions for those experiencing cognitive decline and dementia.

## Ethics Statement

This study was carried out in accordance with the recommendations of the National Health and Medical Research Council’s National Statement on Ethical Conduct in Human Research (2007), the Australian Code for the Responsible Conduct of Research (2007), and the Macquarie University Code for the Responsible Conduct of Research. All subjects gave written informed consent in accordance with the Declaration of Helsinki. The protocol was approved by Macquarie University’s Human Research Ethics Committee and the Management Committee of the Australian Imaging, Biomarkers, and Lifestyle (AIBL) Study of Ageing.

## Author Contributions

AB, CH, TM, and GS developed and designed the study. GS facilitated access to the AIBL participants. TM and AB led the conduct of the study and data collection assisted by CH and two research assistants. CH led the statistical analysis assisted by AB and TM. AB and CH drafted the manuscript with contributions from TM. Finally, all authors contributed to manuscript revision and all authors read and approved the submitted version.

## Conflict of Interest Statement

The authors declare that the research was conducted in the absence of any commercial or financial relationships that could be construed as a potential conflict of interest.
